# Risk Factors of Silicosis Progression: A Retrospective Cohort Study in China

**DOI:** 10.3389/fmed.2022.832052

**Published:** 2022-04-04

**Authors:** Hua Quan, Wenhong Wu, Guanghong Yang, Yunlin Wu, Wenlan Yang, Chunyan Min, Jinyun Shi, Lianhua Qin, Jin Huang, Jie Wang, Xiaochen Huang, Ling Mao, Yonghong Feng

**Affiliations:** ^1^Key Laboratory of Environment Pollution Monitoring and Disease Control, Ministry of Education, School of Public Health, Guizhou Medical University, Guiyang, China; ^2^Department of Pneumoconiosis, Shanghai Pulmonary Hospital, Tongji University, Shanghai, China; ^3^Shanghai Key Laboratory of Tuberculosis, Shanghai Pulmonary Hospital, Tongji University, Shanghai, China; ^4^Guizhou Provincial Center for Disease Control and Prevention, Guiyang, China; ^5^Department of Pulmonary Function Test, Shanghai Pulmonary Hospital, Tongji University, Shanghai, China; ^6^The Fifth People’s Hospital of Suzhou, Suzhou, China; ^7^Department of Radiology, Shanghai Pulmonary Hospital, Tongji University, Shanghai, China

**Keywords:** artificial stone, complicated silicosis, HRCT, lung function, progression

## Abstract

**Background:**

Silicosis poses a threat to workers’ health due to the irreversible lung lesions.

**Design:**

A retrospective cohort study.

**Methods:**

A total of 259 patients [80 worked with artificial stone (AS), 179 with non-artificial stone (non-AS)] with confirmed silicosis were included in this study. Forty-one of AS and 91 of non-AS had approximately 2 years’ follow-up records [lung function tests and high-resolution computer tomography (HRCT)]. Compared with the first records, increased, densified, or newly emerging lesions in lung HRCT images were judged as progression of the disease. Cox proportional hazards models were used to determine the risk factors. Kaplan–Meier survival curve and log-rank test were used to compare prognostic factors for cumulative risk of progression.

**Results:**

In 132 patients with median follow-up of 24.0 months (IQR, 13.8, 24.9), 66 patients showed progression, in them, 36 (87.8%) were from AS group and 30 (32.9%) from non-AS group. Working experience of AS processing (hazard ratio, 5.671; 95% CI, 3.048–10.550) and complicated silicosis in CT images (hazard ratio, 2.373; 95% CI, 1.379–4.082) were the main risk factors associated with progression. Forced vital capacity decreased after 1-year (241.5 vs. 55.2 mL) and 2-year (328.1 vs. 68.8 mL) follow-up in the two groups (AS vs. non-AS). History of anti-tuberculosis medication, chest oppression and pain, ground-glass opacity, pleural abnormalities, and restrictive pulmonary dysfunction were more frequently found on HRCT images in the AS group than non-AS group. Lung functions (DL_CO_, %) were lower in the current/former smokers than the non-smokers (*P* < 0.05) in AS patients.

**Conclusion:**

Prevention and protection rules are needed to be enforced in the occupation involving AS processing; smoking may be associated with declined lung function in AS patients.

## Introduction

Silicosis caused by inhalable respirable crystalline silica, is a worldwide occupational lung disease (1); the progression of pulmonary lesions accompanied with cough, expectoration, chest oppression, and shortness of breath, leading to lethal fibrosis (2). Silicosis is widely prevalent in those who working in mining, quarrying, cutting, and polishing (3); it kills more than 10,000 people every year in the world (4), mainly in developing countries (5). According to a report based on data from Global Burden of Disease Study 2017 (6), the overall age-standardized incidence rate of silicosis decreased by an average of 0.8% per year in 1990–2017 globally.

However, in recent years, silicosis has become an issue of concern, due to the processing of artificial stone (AS). AS materials have a higher silica content (>90%) when compared with natural alternatives (2–30%) (7). It has been found that the time of occupational exposure in AS-associated silicosis cases was less, but progression of the disease was faster than classical silicosis (8, 9).

Up to now, there is no report on risk factors for the cumulative progression in silicosis. Previous studies have shown that high-resolution computer tomography (HRCT) has higher sensitivity in detecting pulmonary nodular changes [including progressive massive fibrosis (PMF), pulmonary bullae, emphysema, and changes in pleura and mediastinal hilum] (10–13). In this study, we collected medical information of patients and focused on the cases with around 2-year follow-up records of HRCT and respiratory function tests. We combined HRCT data with indices of lung function for evaluating progress of the disease (14). This is the first report to compare the cumulative progression rate between patients with artificial stone-associated silicosis and non-artificial stone-associated silicosis.

## Materials and Methods

### Study Population and Procedures

From April 2011 to April 2021, a total of 432 male native Chinese with silicosis who visited the Pneumoconiosis Department of Shanghai Pulmonary Hospital were included in the retrospective cohort study. All the patients left the previous dust environment after being diagnosed as silicosis. We collected all the electronic medical records of the patients and set up a database, which included information such as age at diagnosis of silicosis, age at first dust exposure, years of dust exposure, time from dust exposure to illness, smoking status, respiratory symptoms, indices for respiratory function, and HRCT radiographs of the chest.

Exclusion criteria were: (1) cases with active pulmonary tuberculosis, non-tuberculous mycobacteria (NTM) infection, lung tumor, respiratory infection, pneumothorax, pleural effusion, asthma, and bronchiectasis at the time of first visit; (2) patients without lung function and chest HRCT tests; (3) patients without the information of dust exposure; (4) patients who reject taking part in this study.

**FIGURE 1:**
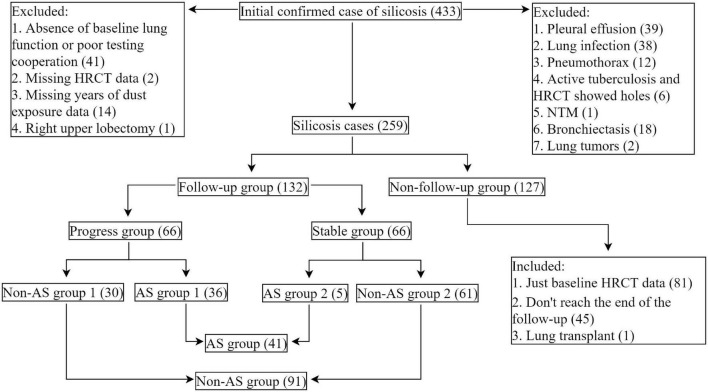
Study flowchart. Cases of silicosis in Shanghai Pulmonary Hospital, Shanghai, China (2011.04–2021.04). AS-associated silicosis group is referred to as AS group; non-AS-associated silicosis group is referred to as non-AS group.

After exclusion, 259 patients were left, in which 132 patients were with HRCT records in about 2-year follow-up periods (Figure 1).

### Respiratory Function and High-Resolution Computer Tomography Tests

Respiratory function tests were performed according to the ATS/ERS recommendations and measured with a clinical spirometer (Jaeger Crop., Höchberg, Germany) by specialists from the department of the pulmonary function in Shanghai Pulmonary Hospital (15–17). The main ventilatory pulmonary function indicators (18, 19) including forced vital capacity (FVC, %), forced expiratory volume in 1 s (FEV_1_, %), FEV_1_/FVC ratio, and diffusing capacity of the lung for carbon monoxide (DL_CO_, %) were analyzed. Meanwhile, according to the prediction model of Wells et al. (20), we calculated the compound physiological index (CPI). The calculation formula is as follows: CPI = 91.0 – (0.65 × DL_CO_, %) – (0.53 × FVC, %) + (0.34 × FEV_1_, %).

All patients underwent HRCT and respiratory function tests upon admission. According to the size of the mass in the HRCT image, patients were divided into simple silicosis group and complicated silicosis group. Complicated silicosis is defined by the presence of nodules measuring 1 cm or more (10, 11, 21, 22). The increase and densification of lesions, or newly emerging lesions, are defined as progression (12), and stability is defined as no significant change of HRCT manifestations at least 22 months. The diagnosis of patients was made by two qualified physicians from the pneumoconiosis department and HRCT images were read by two experienced doctors from radiology department. According to the comparison results, patients were then grouped into the stable group (stable in 22.1–32.6 follow-up months) and progressive group (progress in 1.1–35.9 follow-up months).

### Statistical Analysis

Cox proportional hazards models were used to determine the risk factors for progressing of disease according to HRCT imaging. The Kaplan–Meier survival curve and log-rank test were used to compare prognostic factors with a cumulative risk of progression over time. The epidemiological and clinical variables between the two groups are expressed as Means ± standard deviation (SD), Median (interquartile range, IQR), and percent of individuals. The Student’s *t*-test, Mann–Whitney *U*-test, Chi-square test, or Fisher’s exact test were used to evaluate differences between the two groups, as appropriate. All data analyses were conducted using SPSS version 25.0 (IBM SPSS, Chicago, IL, United States) with the prominence level set to 5%.

## Results

### Comparison of Baseline Characteristics of Silicosis Patients

The total of 259 patients were divided into AS group (80 patients) and non-AS group (179 patients) according to their working history with artificial stone. We first compared the baseline data between the AS and the non-AS, the latter one included 66 patients with working history of quarrying, 15 patients with coal mining, 28 cases with sand blasting, 13 cases with granite fabrication, 21 cases with refractory, 9 cases with tunneling, 15 cases with metal mining, and 12 cases with other types of work.

The median age at diagnosis of silicosis in AS group [35.5 years (IQR, 29.9, 46.4)] is younger than non-AS group [51.4 years (IQR, 45.5, 58.9)] and with less time of dust exposure [7.0 years (IQR, 5.0, 8.0) vs. 18.00 years (IQR, 10.0, 27.0)]. The shortest time of dust exposure among all patients was only 1.5 years (in the AS group) and the longest was 43 years (in the non-AS group) (all *P* < 0.05) (Table 1).

**TABLE 1:** Demographic characteristics and HRCT features of AS group versus non-AS group.

Characteristics	AS group (*n* = 80)	Non-AS group (*n* = 179)	*P*-value
**Demographic characteristics^a^**			
Age at diagnosis of silicosis, years	35.5 (29.9, 46.4)	51.4 (45.5, 58.9)	**<0.001**
Age at onset of dust exposure, years	28.0 (22.0, 37.5)	23.0 (17.0, 23.0)	**<0.001**
Years of dust exposure, years	7.0 (5.0, 8.0)	18.0 (10.0, 27.0)	**<0.001**
Time from dust exposure to illness, years	7.0 (4.9, 9.5)	25.3 (17.6, 35.2)	**<0.001**
Current/former smoker, n (%)^b^	32 (40.0)	64 (35.8)	0.513
History of anti-tuberculosis treatment, n (%)^b,c^	9 (11.2)	8 (4.4)	**0.042**
Complicated silicosis, n (%)^b^	26 (32.5)	51 (28.5)	0.514
**HRCT features^a^**			
Appears with clinical symptoms, n (%)	60 (72.3)	155 (92.9)	**<0.001**
Cough and expectoration, n (%)	40 (50.0)	141 (78.8)	**<0.001**
Chest oppression and pain, n (%)	43 (53.7)	69 (38.5)	**0.023**
Mass shadow, n (%)	31 (38.8)	55 (30.8)	0.205
Pleural abnormalities, n (%)	34 (42.5)	43 (24.0)	**0.003**
GGO, n (%)	21 (26.3)	7 (3.9)	**<0.001**
Mediastinal and hilar lymphadenopathy, n (%)	55 (68.8)	126 (70.4)	0.790

*^a^P-value from Mann–Whitney U-test, data are presented as median (IQR) unless otherwise indicated. ^b^P-value from Chi-square test, data are presented as percent of individuals. ^c^Patient received preventive anti-tuberculosis treatment 2 years before the first admission. GGO, ground-glass opacity. Significant p-values (P < 0.05) are provided in bold.*

The age of first dust exposure in the AS group was older than that in the non-AS group. The ratio of patients with a history of anti-tuberculosis treatment in the AS group (11.2%) was higher than those in the non-AS group (4.4%). The median time from the dust exposure to diagnose as silicosis in the AS group was 7.0 years (IQR, 4.9, 9.5), significantly shorter than 25.3 years (IQR, 17.6, 35.2) in the non-AS group (all *P* < 0.05) (Table 1).

As sandblasting is also associated with severe silicosis and accelerated progress of the disease (23, 24), we also compared the characteristics of the 28 sand-blasting workers with 151 other workers in the non-AS group (non-AS group 3), and with those of AS patients. As shown in Supplementary Tables 1, 2, patients with history of sand-blasting were at the similar ages at diagnosis of silicosis with the other patients in non-AS groups. The years of dust exposure and time from dust exposure to illness in the sand-blasting patients were between those of AS group (*P* < 0.05) and of non-AS group (*P* < 0.05).

In the 259 patients, 13.8% (27.7% in the AS group and 7.1% in the non-AS group) patients only had mass shadows in lung on HRCT images in previous physical examination without clinical symptoms. There were more patients with cough and expectoration, chest oppression and pain, ground-glass opacity, and pleural abnormalities in the AS group than in the non-AS group (all *P* < 0.05), no significant difference were found between the groups on ratios of mass shadow and mediastinal and hilar lymphadenopathy (Table 1).

### Lung Function in Patients at Baseline

**FIGURE 2:**
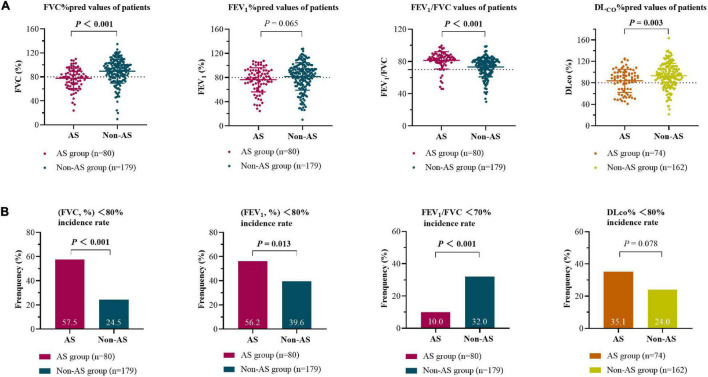
Baseline lung function (measured/predicted) for silicosis with AS group and non-AS group **(A)** and the incidence of pulmonary dysfunction in the two groups **(B)** at the first registration. The area above the dotted line represent the normal ranges of the indices. The differences between groups were analyzed by Student’s *t*-tests **(A)** and Chi-square tests **(B)**. FVC, forced vital capacity; FEV_1_, forced expiratory volume in 1 s; DL_CO_, diffusing capacity of the lung for carbon monoxide.

In the AS group, the baseline average values of FVC (%) and FEV_1_ (%) of patients were decreased (the normal values of the two indices are >80.0%), while the average of FVC (%), FEV_1_ (%), FEV_1_/FVC, and DL_CO_ (%) were all within the normal ranges in the non-AS group (Figure 2). Restrictive pulmonary dysfunction (FVC, % <80.0%) were observed in 57.5 and 24.5% of the two groups, respectively (*P* < 0.001), while obstructive pulmonary dysfunction (FEV_1_/FVC < 70.0%) occurred in 10.0 and 32.0% of patients (*P* < 0.001), and diffusion dysfunction (DL_CO_, % <80.0%) occurred in 35.1 and 24.0% of patients (*P* = 0.078). Also, there is a significant difference with FEV_1_ in the two groups (*P* < 0.05).

The CPI score calculated by respiratory function tests in the AS group at initial evaluation were higher than those in the non-AS group (Table 2).

**TABLE 2:** Baseline lung function characteristics with AS group versus non-AS group.

Variable	AS group (*n* = 80)			Non-AS group (*n* = 179)			*P*-value^b^
	Total (*n* = 80)	Current/former smoker (*n* = 32)	Non-smoker (*n* = 48)	Total (*n* = 179)	Current/former smoker (*n* = 64)	Non-smoker (*n* = 115)	
FVC, %	77.7 ± 17.0	74.3 ± 17.2	80.0 ± 16.6	89.4 ± 19.8	88.2 ± 21.2	90.0 ± 19.1	**<0.001**
FEV_1_, %	76.3 ± 20.0	**70.9 ± 20.4**	**79.7 ± 19.0^c^**	81.7 ± 22.7	80.2 ± 23.2	82.5 ± 22.4	0.065
FEV_1_/FVC, %	81.4 ± 10.8	78.9 ± 10.9	82.9 ± 10.5	73.1 ± 12.2	72.2 ± 12.2	73.5 ± 12.2	**<0.001**
DL_CO_, %^a^	83.7 ± 21.6	**76.8 ± 21.9**	**88.4 ± 20.3^d^**	93.2 ± 23.1	91.3 ± 23.2	94.3 ± 23.2	**0.003**
CPI scores	21.1 ± 16.2			10.3 ± 16.3			**<0.001**

*^a^Data from 74 patients in AS group (current/former smoker n = 30, non-smoker n = 44) and 162 patients in non-AS group (current/former smoker, n = 59, non-smoker, n = 103). ^b^P-value from Paired Student’s t-test between data from total patients of AS groups and non-AS group, data are presented as mean ± SD. ^c^P = 0.053, ^d^P = 0.022 compared between current/former smoker and non-smoker. FVC, forced vital capacity; FEV_1_, forced expiratory volume in 1 s; DL_CO_, diffusing capacity of the lung for carbon monoxide; CPI, composite physiological index. Significant p-values (P < 0.05) are provided in bold.*

The current or former smokers had statistically lower values of DL_CO_ (%) (*P* = 0.022) or tendency of lower FEV_1_ values (*P* = 0.053) in AS group but not in non-AS group (Table 2).

### Lung Function in Patients at 0-to-1-Year and 0-to-2-Year Follow-Up

**FIGURE 3:**
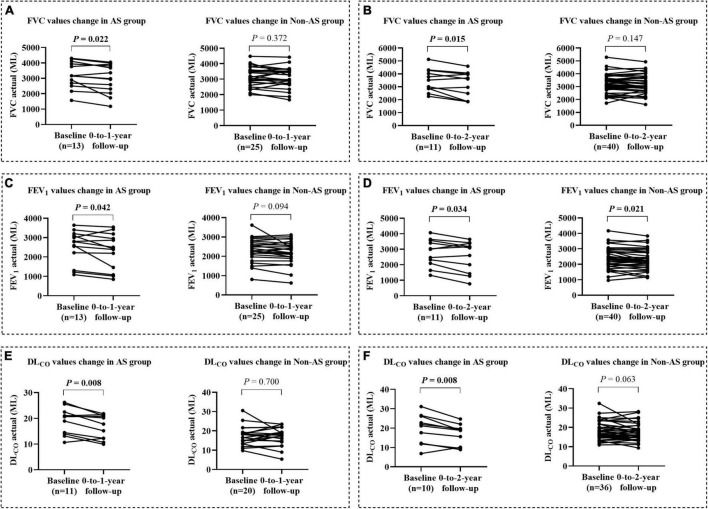
The changes of lung function from baseline to the values at 0-to-1-year and 0-to-2-year followed up in AS and non-AS groups. Changes of FVC, FEV_1_, and DL_CO_ (mL) from baseline to the values after 1 **(A,C,E)** and 2 years **(B,D,F)** follow-up. FVC, forced vital capacity; FEV_1_, forced expiratory volume in 1 s; DL_CO_, diffusing capacity of the lung for carbon monoxide.

We collected and compared the data of respiratory function tests from patients of AS group and non-AS group with complete records during 0-to-1-year (AS, *n* = 13, non-AS, *n* = 25) and 0-to-2-year (AS, *n* = 10, non-AS, *n* = 26) follow-up. The results showed that the average FVC, FEV_1_, and DL_CO_ in the AS group were all significantly decreased at either 1 year (Figures 3A,C,E) or 2 years (Figures 3B,D,F) compared with the baseline records; while only average FEV_1_/FVC in the non-AS group showed a significant decrease in both 1- and 2- years follow-up tests (data not shown). The lung function indices shown as percentages of the predicted values had similar changes in the two groups as the changes of the actual values (Supplementary Figures 1A–F).

### Risk Factors in the Progression of Silicosis

The progression was used as follow-up endpoint in the analysis of follow-up records from 132 patients with median follow-up time of 24.0 months (IQR, 13.8, 24.9). During the follow-up, progression occurred in 66 patients (50.0%).

**FIGURE 4:**
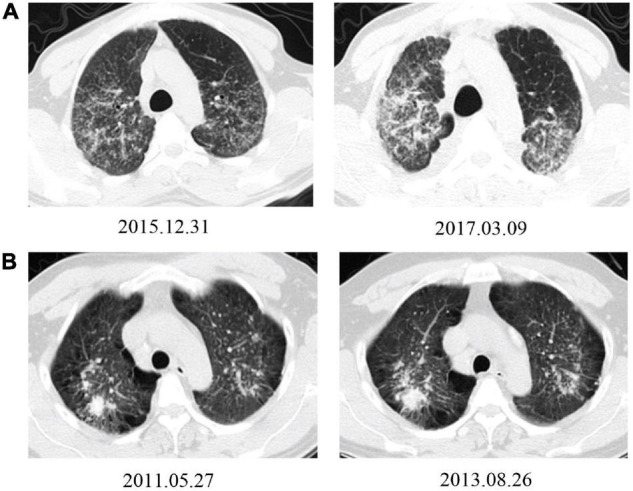
Typical HRCT images show progression from patients working with AS and non-AS. **(A)** A 36-year-old man had been working in AS cutting and home installing for 6 years. The HRCT image from the first registration (upper-left) and rapid progression after 15 months follow-up, with an increase of small nodules in both lung lobes; some of them connected into pieces, with pleural adhesions (upper-right). **(B)** A 40-year-old man had been working in metal mining for 7 years. The HRCT images from the first registration (lower-left) and slow radiological progression after 27 months follow-up period with an slightly enlargement in the upper right lung mass, increment in emphysema and bullae, enlargement and calcification in mediastinal lymph nodes (lower-right).

The association between the progression with working experience of AS processing, complicated silicosis in CT images, years of dust exposure, baseline FVC (%), age at diagnosis of silicosis, and smoking status were analyzed by Multivariate Cox proportional hazards models. The results adjusted by working experience of AS processing and/or age at diagnosis of silicosis showed that patients with working experience of AS processing (hazard ratio, 5.671; 95% CI, 3.048–10.550) and with complicated silicosis (hazard ratio, 2.373; 95% CI, 1.379–4.082) had significantly higher risks of progression during the follow-up periods (all *P* < 0.01) (Table 3 and Figure 4).

**TABLE 3:** Factors associated with silicosis progress in multivariate Cox proportional hazards model^a^.

	Unadjusted			Adjusted^c^		
	HR	95% *CI*	*P*-value	HR	95% *CI*	*P*-value
Working experience of AS processing (yes)	4.422	2.688–7.274	**<0.001**	5.671	3.048–10.550	**<0.001**
Complicated silicosis	1.786	1.057–3.017	**0.030**	2.373	1.379–4.082	**0.002**
Age at diagnosis of silicosis	0.977	0.957–0.997	**0.023**	1.016	0.993–1.039	0.184
Baseline FVC (%)	0.998	0.997–0.999	**0.038**	0.996	0.982–1.009	0.519
Smoking status (current/former)^b^	1.243	0.740–2.090	0.411	1.221	0.725–2.059	0.453

*^a^The risk factors in the Multivariate Cox proportional hazards models were determined based on clinical experience and the studies of Leon-Jimenez et al. (31). ^b^Patients who had quitted smoking 1–10 years before the first registration were in former smokers, and those who had quitted for more than 10 years were in never smokers. ^c^Estimations were adjusted by working experience of AS processing and/or age at diagnosis of silicosis. AS, artificial stone; FVC, forced vital capacity; HR, hazard ratio; SE, standard error; CI, confidence interval. Significant p-values (P < 0.05) are provided in bold.*

### Comparison of Disease Progression Rates in Silicosis Patients

**FIGURE 5:**
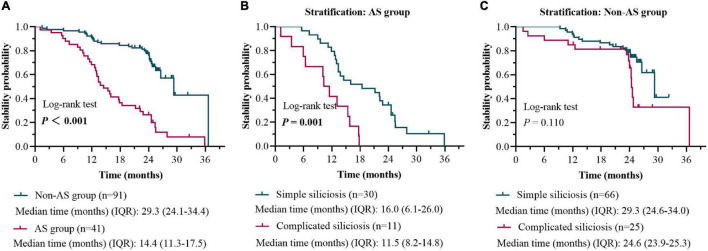
HRCT stability rate in patients with the AS group versus the non-AS group during 0-to-2-year follow-up **(A)**. HRCT stability rate in patients with simple silicosis versus the complicated silicosis in the AS group during 0-to-2-year follow-up **(B)**. HRCT stability rate in patients with simple silicosis versus the complicated silicosis in the non-AS group during 0-to-2-year follow-up **(C)**.

The 132 patients were also sub-grouped into AS group (41 patients) and non-AS group (91 patients) according to their working experience of AS processing. During the follow-up, the disease progression rates of patients in the AS group and the non-AS group were 87.8% (36/41) and 32.9% (30/91), respectively (Figure 5). Among them, 26.6% (8/30) of patients with simple silicosis in the AS group developed PMF, while none developed PMF (0.0%) in the non-AS group.

The Kaplan–Meier survival curve and log-rank test based on the results of Cox proportional hazards models were used to compare the difference of stability probability (1-progress probability) between the AS group and the non-AS group. The median time of stability in the AS group was 14.4 months (IQR, 11.3, 17.5), which was less than 29.3 months (IQR, 24.1, 34.4) in the non-AS group (log-rank, 40.57; *P* < 0.001) (Figure 5).

When patients with or without working experience of AS processing were further stratified as subgroups of simple and complicated silicosis, respectively, a significant difference in time of stability between the subgroups (simple silicosis vs. complicated silicosis) was found only in the patients with AS processing history (*P* < 0.001, Figure 5).

## Discussion

According to the data from the global report (25), the incidence counts of silicosis patients in 2017 was 23,700. However, reported patients are only the tip of the iceberg, particularly in developing countries (21). In recent years, an increasing number of silicosis patients among workers exposed to high amounts of dust (more than 90% of crystalline silica) caused by processing kitchen and bathroom countertops has been found (26–29). The prevalence AS-associated fast-forward silicosis has drawn special attention (30).

It is the first report about the comparison of the cumulative risk of progression between patients with silicosis with two different occupational exposure environments. In this study, we found that the patients in AS group had significantly higher risks of disease progression than patients in the non-AS group after adjusting by common/previous reported progression risk factors (31). We showed that a significant difference in the progression rate between simple and complicated silicosis was found only in the AS group.

During the follow-up times, our results show that working experience of AS processing was the main risk factor for patients in the progression of silicosis, which increased 5.671 folds of risk of progression with a shorter median time of stability in AS patients than in non-AS processing (14.4 months vs. 29.3 months). In a larger cohort study of the miners, patients with PMF increased 9.4% in a 22-year follow-up after dust exposure (32). Recently, Leon-Jimenez et al. (31) from Spain observed 106 newly diagnosed patients with artificial stone-associated silicosis had PMF increased 31.1% after a mean 4-year follow-up. Our study furthered to find that, the ratio of patients with simple silicosis developed PMF increased 26.6% in the AS group, while no patients developed PMF in non-AS group in the follow-up. Consistently, the progress probability in AS group of patients was significantly higher than that in the non-AS group (87.8 vs. 32.9%).

Meanwhile, the AS-associated silicosis is with a sharp decline in lung function. A study from Spain (31) showed a decrease in average FVC of 86.8 mL per year in 106 patients with AS-associated silicosis. Our results showed that after 1-year and 2-year follow-up, average FVC values of patients in the AS group decreased more than those in the non-AS group (241.5 vs. 55.2 mL, 328.1 vs. 68.8 mL). At baseline, lung function in the AS group was also worse than that in the non-AS group (FVC, %: 77.7 ± 17.0 vs. 89.4 ± 19.8; DL_CO_, %: 83.7 ± 21.6 vs. 93.2 ± 23.1). In addition, 57.5% of patients in the AS group with restrictive pulmonary dysfunction, which was similar to the previous report about the most common type of lung function impairment in patients with AS- associated silicosis (33). CPI is commonly used to evaluate the severity of idiopathic pulmonary fibrosis (IPF) disease (20). A higher CPI score in AS group than in non-AS group also indicated that the patients in the AS group had a more serious impairment in lung function on the whole.

The median age at diagnosis of silicosis in the AS group was 35.5 years (IQR, 29.9–46.4), significantly younger than that in the non-AS group. The workers with working experience of AS processing at a younger age have been reported by Hoy et al. (average age 36 years) in Australia and other studies (26, 27). Several studies have reported on time of dust exposure in silicosis patients. Qiao Ye’s team from China (34) reported an average dust exposure time of 6.1 years in 18 patients with AS-associated silicosis. A study conducted in metal mines and pottery factories in China found that the average time of dust exposure in 2,857 silicosis patients was 18.4 years (35). Similar to previous reports, the time of dust exposure of patients in the AS group in our study was 7.0 years (IQR, 5.0–8.0), which was significantly shorter than 18.0 years (IQR, 10.0–27.0) in the non-AS group (36), and the shortest exposure time was only 1.5 years.

In our previous investigation of processing sites for patients with AS-associated silicosis (37), α-quartz content in dust in the air of 5 processing workshops and installation sites were 70–99%, with mass concentrations of (127.6 ± 17.3) mg/m^3^, respectively. It is 255 times higher than the permissible concentration-time weighted average (PC-TWA, <0.5 mg/m^3^) in China. As the patient with AS-associated silicosis is relatively younger and has a shorter time of dust exposure than patients with classical silicosis, more prevention and protection rules are needed to be enforced in this occupational field.

Patients in the AS group were more likely to have chest oppression and pain (53.7%). In addition, the number of patients with the history of anti-tuberculosis treatment is more in AS group than those in non-AS group silicosis (11.2 vs. 4.4%). It may indicate that the imaging manifestations of AS-associated silicosis are similar to tuberculosis at the early stage, and the differential diagnosis may be more difficult than those in the non-AS group. In terms of imaging, our study found that patients were more likely to appear pleural abnormalities and ground-glass opacity in the AS group than those in the non-AS group, which were similar to a previous report from China (34).

The impact of smoking on silicosis is still controversial. Smoking was considered a risk factor for silicosis in earlier studies (38), but some study reported that there was no significant association between silicosis and smoking status (39). Previous data (34) indicated that there was no significant difference in the effects of smoking on lung function between the artificial stone-associated silicosis and natural stone-associated silicosis. However, our study indicated that, in the AS group, smoking is associated with reduced DL_CO_ (%) value, and may be reduced FEV_1_ value too (*P* = 0.053). Although no impact was found in smoking status on progression of the disease, it may be a risk factor for decreased lung function in the AS group.

In addition, sandblasting workers as fast-forward silicosis but in non-AS group in this study caught our attention. The 28 patients in sand-blasting sector were older than AS sector, but were at the similar ages of the other non-AS patients. The years of dust exposure and the time from dust exposure to illness in sand-blasting sector were longer than those in AS sector but less than other patients in non-AS sector. The progress in sand-blasting cases were slower than AS cases, however, showed a tendency of 2–3 folds faster than that in non-AS cases but without statistical significance. Small size of the sand-blasting sector might be one reason, as 5 in these 28 cases did sieving work thus may exposure to less concentration of dust.

We also compared some of our results in the AS group with the reported data in sandblasting. In one study (40), CT findings in 50 male patients with denim sandblasters were evaluated. Pleural thickening was positive in 19 cases (38%), similar to our result in AS group (42.5%). In another report (41), the ages at first admission in 83 living man participants (96.4% of them had been diagnosed with silicosis) were 23 ± 6 years, and the exposure duration were 41 ± 27 months. The exposure duration in the report is much shorter than 11.0 (6.0, 18.0) years in the 28 sand-blasting in our study.

In tracking the background of the 28 sand-blasting workers, 21 were found from state-owned enterprises in Shanghai and 7 from other areas in China. Protective equipment and measures normally can be available by state-owned enterprise workers, therefore the concentration of dust in their working environment might be far less than that in the environment of artificial stone cutting, and the disease progress relatively slower than AS cases. However, the comparison between sandblasting and AS-associated silicosis in China need more data before reaching a conclusion.

As this is a retrospective study, some drawbacks may exist. For example, few patients had lung function tests during the follow-up period, therefore we were unable to explore the correlation between smoking, the decline in lung function and the progression of HRCT, especially in AS group. In addition, the data were from a single medical center and a lack of long-term follow up from the patients also caused the limitation to our research.

## Conclusion

Patients with the AS-associated silicosis had more than 5 folds higher risk of developing progression with a significant decline in lung function than the patients from the non-AS group during a 2-year follow-up. Complicated silicosis progresses faster than simple silicosis only occurred in the AS group. More evidence is needed to determine whether smoking status will increase the progressing incidence of AS-associated silicosis.

## Data Availability Statement

The raw data supporting the conclusions of this article will be made available by the authors, without undue reservation.

## Ethics Statement

The studies involving human participants were reviewed and approved by the Institutional Review Board of Tongji University (project approval number: K18-142). The patients/participants provided their written informed consent to participate in this study. Written informed consent was obtained from the individual(s) for the publication of any potentially identifiable images or data included in this article.

## Author Contributions

HQ, YF, and LM applied conception, designed the research, and wrote the article. HQ, WW, JW, JH, LQ, XH, and YW collected the clinical data. CM, WY, and LM interpreted the lung function data. LM, JS, and CM interpreted the radiologic data. HQ, WW, YW, LM, and YF analyzed and interpreted the clinical data. YF and GY provided financial support fund and conducted the entire research. All authors read and approved final manuscript.

## Conflict of Interest

The authors declare that the research was conducted in the absence of any commercial or financial relationships that could be construed as a potential conflict of interest.

## Publisher’s Note

All claims expressed in this article are solely those of the authors and do not necessarily represent those of their affiliated organizations, or those of the publisher, the editors and the reviewers. Any product that may be evaluated in this article, or claim that may be made by its manufacturer, is not guaranteed or endorsed by the publisher.

## Supplementary Material

Supplementary Figure 1 The changes of lung function indices (% predicted values) from baseline to the values at 0-to-1-year and 0-to-2-year followed up in AS and non-AS groups. Changes of FVC, FEV_1_, and DL_CO_ (% predicted values) from baseline to the values after 1 **(A,C,E)** and 2 years **(B,D,F)** follow-up. FVC, forced vital capacity; FEV_1_, forced expiratory volume in 1 s; DL_CO_, diffusing capacity of the lung for carbon monoxide.

Supplementary Table 1 Demographic characteristics of AS group versus sandblast group and Non-AS group versus sandblast group.

Supplementary Table 2 Factors associated with silicosis progress in multivariate Cox proportional hazards model.
