# Supplementary figures and images for: Risk Factors of Silicosis Progression: A Retrospective Cohort Study in China
Source: Front Med (Lausanne). 2022 Apr 4;9:832052. doi: 10.3389/fmed.2022.832052 (PMC9013759; doi:10.3389/fmed.2022.832052)

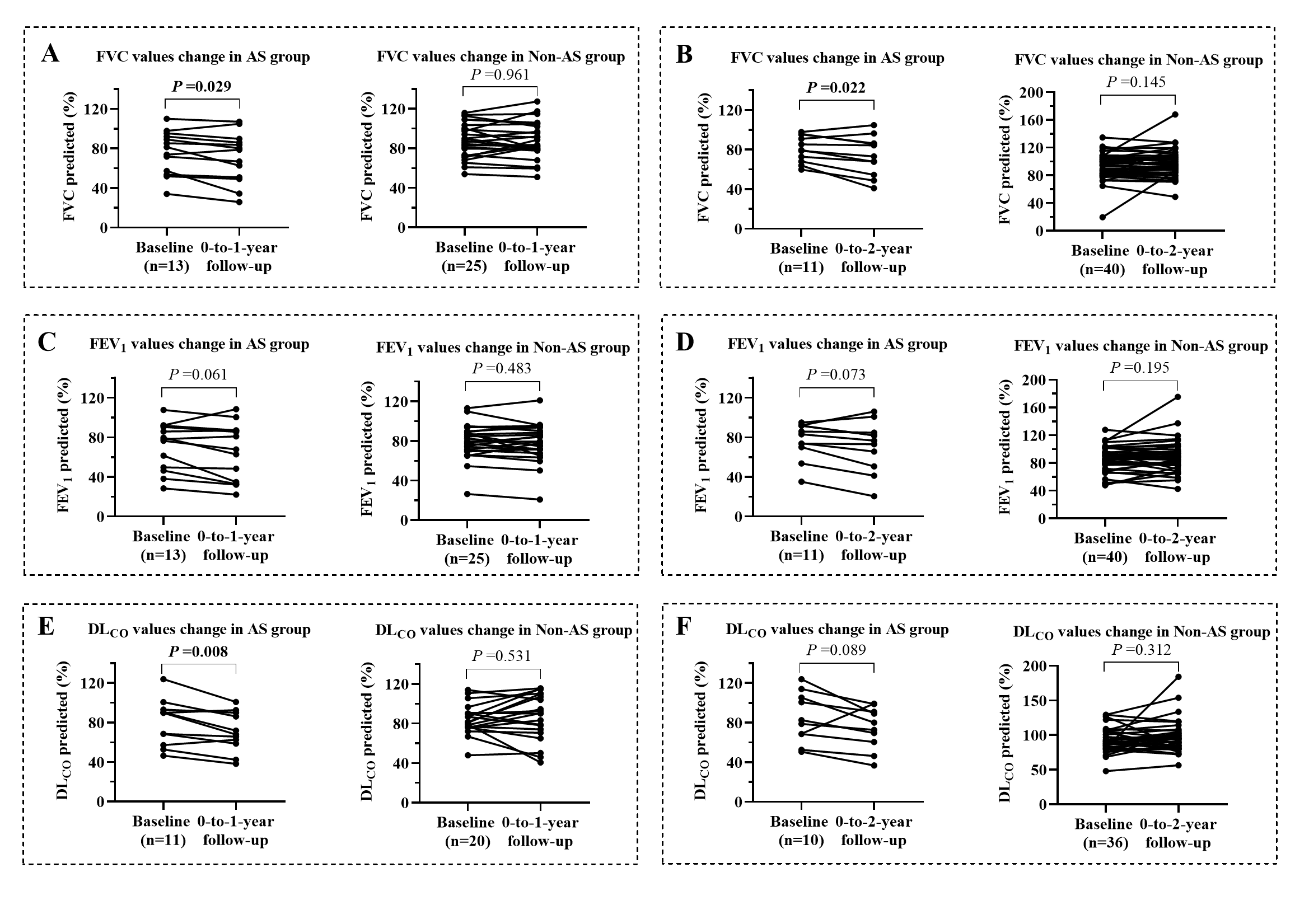

Supplement: Supplementary Figure 1 — The changes of lung function indices (% predicted values) from baseline to the values at 0-to-1-year and 0-to-2-year followed up in AS and non-AS groups. Changes of FVC, FEV1, and DLCO (% predicted values) from baseline to the values after 1 (A,C,E) and 2 years (B,D,F) follow-up. FVC, forced vital capacity; FEV1, forced expiratory volume in 1 s; DLCO, diffusing capacity of the lung for carbon monoxide. [file Image_1.TIF]
