# Supplementary material for: Risk Factors of Silicosis Progression: A Retrospective Cohort Study in China
Source: Front Med (Lausanne). 2022 Apr 4;9:832052. doi: 10.3389/fmed.2022.832052 (PMC9013759; doi:10.3389/fmed.2022.832052)
Supplement: Supplementary Table 1 — Demographic characteristics of AS group versus sandblast group and Non-AS group versus sandblast group. [file Table_1.docx]

**Supplementary TABLE 1 |** Demographic characteristics of AS group versus sandblasting group and Non-AS group 3 versus sandblasting group

| **Demographic characteristics** ^a^ | **AS group**  **(n = 80)** | **Sandblasting**  **group (n =28)** | ***P*-Value** | **Non-AS group 3 (n = 151)** | **Sandblasting**  **group (n =28)** | ***P*-Value** |
| --- | --- | --- | --- | --- | --- | --- |
| Age at diagnosis of silicosis, years | 35.5 (29.9, 46.4) | 51.4 (44.0, 59.1) | **<0.001** | 51.4 (45.5, 58.9) | 51.4 (44.0, 59.1) | 1.000 |
| Age at onset of dust exposure, years | 28.0 (22.0, 37.5) | 29.0 (22.0, 37.0) | 1.000 | 23.0 (17.0, 23.0) | 29.0 (22.0, 37.0) | **0.007** |
| Years of dust exposure, years | 7.0 (5.0, 8.0) | 11.0 (6.0, 18.0) | **0.004** | 18.0 (10.0, 27.0) | 11.0 (6.0, 18.0) | **0.001** |
| Time from dust exposure to illness, years | 7.0 (4.9, 9.5) | 18.3 (8.3, 35.4) | **<0.001** | 25.3 (17.6, 35.2) | 18.3 (8.3, 35.4) | **0.040** |

*^a^ P-Value from Mann-Whitney U-test, data are presented as median (IQR) unless otherwise indicated. Values in bold signify P<0.05.*

*AS, artificial stone; AS group, artificial stone; Non-AS group, non-artificial stone; P, probability.*

| **Supplementary TABLE 2 \|** Factors associated with silicosis progress in multivariate Cox proportional hazards model ^a^. | | | | | | | |
| --- | --- | --- | --- | --- | --- | --- | --- |
| **Groups** | Unadjusted | | |  | Adjusted ^b^ | | |
|  | **HR** | **95%*CI*** | ***P*-value** |  | **HR** | **95%*CI*** | ***P*-value** |
| AS vs sandblasting | 9.608 | 2.300-40.143 | **0.002** |  | 11.331 | 2.566-50.034 | **0.001** |
| Sandblasting vs non-sandblasting | 2.752 | 0.653-11.594 | 0.168 |  | 2.703 | 0.641-11.407 | 0.133 |

*^a^ The risk factors in the Multivariate Cox proportional hazards models were determined based on clinical experience and the studies of*

*Leon-Jimenez et al (Ref. 31).*

*^b^ Estimations were adjusted by working experience of AS processing and / or age at diagnosis of silicosis.*

*AS, artificial stone; HR, hazard ratio; SE, standard error; CI, confidence interval.*
